# Process and Anti-Mildew Properties of Tea Polyphenol-Modified Citral-Treated Bamboo

**DOI:** 10.3390/molecules27217549

**Published:** 2022-11-03

**Authors:** Shiqin Chen, Qi Li, Chunlin Liu, Chungui Du, Yingying Shan, Wenxiu Yin, Fei Yang, Yuran Shao, Yuting Wang

**Affiliations:** College of Chemistry and Materials Engineering, Zhejiang A&F University, Hangzhou 311300, China

**Keywords:** citral, tea polyphenol, modification, bamboo, anti-mildew

## Abstract

In order to reduce the oxidative degradation of citral, our research group modified citral with the natural antioxidant from tea polyphenols and applied it to bamboo processing to enhance the anti-mold effect of bamboo, but its application to the bamboo treatment process and the anti-mold effect is still not clear. For this reason, in this paper, the tea polyphenol-modified citral anti-mildew treatment of bamboo as well as the anti-mildew properties of bamboo were explored using the orthogonal testing method and a UV-vis spectrophotometer. The results showed that when the concentration of tea polyphenol-modified citral reached 175 mg mL^−1^ and above, the efficacy of the anti-mildew treated bamboo against common molds reached 100%; the improved anti-mildew treatment process parameters for bamboo were as follows: impregnation pressure 0.6 MPa, impregnation time 150 min, and tea polyphenol-modified citral concentration 200 mg mL^−1^. Following the tea polyphenol-modified citral anti-mildew treatment of bamboo, not only did it improve the anti-mildew properties of the bamboo materials, but it also added a fresh lemon fragrance without altering the original colour, microstructure, and chemical properties of the bamboo materials.

## 1. Introduction

As one of the fastest growing plants in the world, bamboo has the advantages of being biodegradable, renewable, having a high tensile strength, good toughness, and low cost [1,2,3,4]. As a result, bamboo and its products are used extensively in the fields of building, furniture, and gardening [5,6,7]. However, because of the hydrophilic and nutrient-rich nature, bamboo and its products have a low natural resistance to mold and mildew [8] and are subject to mildew during use to the extent that they lose their use value and are discarded, leading to significant wastage of resources and economic loss. Consequently, it is of great importance to carry out research into the protection of bamboo against mold. In recent years, bamboo anti-mildew research has become a hot spot in which natural antimicrobial agent-related research has gradually gained attention. One of the natural antimicrobial agents is citral, which is primarily derived from the essential oil of the Chinese medicine Cyperus rotundus, which has the advantages of a broad spectrum of bacterial inhibition and being environmentally friendly and will have a greater outlook for applications in the anti-mildew of bamboo [9,10,11,12,13]. Currently, the study on the inhibition performance of citral against common bamboo molds carried out by the authors’ team [14,15] found that the inhibition rate of bamboo molds exceeded 100% when the concentration of citral was 75 mg mL^−1^. Citral, on the other hand, is unstable and susceptible to oxidative degradation leading to lower inhibition properties [16,17], which limits the application of citral in bamboo materials. Consequently, the antioxidant modification of citral to enhance its antibacterial properties is important to promote the widespread application of citral in the prevention of mold in bamboo.

The modification of citral by adding antioxidants to it is an effective method to improve the stability of citral [17,18,19]. The use of citral as a feedstock and the addition of antioxidants is an effective way to improve citral stability. Tea polyphenols are a type of natural antioxidant extracted from plants. Their advantages include strong antioxidant resistance, broad sources, and high safety [20,21,22]. Research into the synergistic effects of tea polyphenols as antioxidants is of increasing importance [23,24,25,26]. To this end, the authors conducted a preliminary exploration of the preparation of tea polyphenol-modified citral by compounding tea polyphenols with citral and found that tea polyphenol-modified citral had better antioxidant properties when tea polyphenols were added at 1% of the mass of citral. To date, no relevant research reports on the antioxidant-modified citral anti-mildew treatment of bamboo have been reported. Accordingly, this article will conduct a detailed investigation of the process and anti-mildew properties of the tea polyphenol-modified citral anti-mildew treatment of bamboo with the goal of providing a theoretical reference for the promotion and application of citral in the field of bamboo anti-mildew treatment.

## 2. Results and Analysis

### 2.1. Effect of the Tea Polyphenol-Modified Citral Anti-Mildew Treatment Process on the Drug Loading of Bamboo Strips

The effect of the anti-mildew treatment process parameters of bamboo strips, such as impregnation pressure, impregnation time, and concentration of tea polyphenol-modified citral, on the drug loading of bamboo strips was investigated by the orthogonal test method. Table 1, Table 2 and Table 3 show the results of the tests and the analysis of variance (ANOVA), respectively.

The X_i_ value in the table indicates the average value, and X_i_ indicates the effect of each level of factor i on the wet drug loading, R_1_, and dry drug loading, R_2_, of bamboo slices, and the larger the value, the higher the wet drug loading and the dry drug loading of this factor value. X_max_ denotes the maximum value among the three X_i_ values of each factor, X_min_ is the minimum value, and the difference between them is the extreme difference R. The greater the R value, the greater the influence of the factor on the wet and dry drug loading of bamboo. From Table 1 of the orthogonal experimental results, it can be seen that the order of influence of the three factors A, B, and C was A > C > B when using R_1_ as an index of experimental investigation, the optimal combination of conditions for treating bamboo strips was A_2_B_3_C_3_, the impregnation pressure was 0.6 MPa, the pressurized impregnation time was 150 min, the antioxidant citral concentration was 200 mg mL^−1^, and the R_1_ value was 39.78 g/m^2^. The optimal combination of conditions for treating bamboo strips was A_2_B_3_C_3_, an impregnation pressure of 0.6 MPa, a pressure soak time of 150 min, and a concentration of tea polyphenol-modified citral of 200 mg mL^−1^, whereas the R_2_ was 24.98 g/m^2^ under these conditions.

Table 2 shows the ANOVA results on wet R1 drug loading. From Table 2, we can see that at the 10% level of significance, there was a significant difference in the effect of the impregnation pressure on the wet loading of the bamboo material, and this was the main factor. The difference in the effect of the impregnation time and antioxidant lemon concentration on the wet loading of the bamboo material was not significant and was a minor factor. The ANOVA results on the dry drug loading R_2_ are presented in Table 3. From Table 3, we can see that at the 10% significance level, the difference in the effect of the impregnation pressure on the dry loading of bamboo was significant and was the major factor, although the difference in the effect of the impregnation time and antioxidant lemon concentration on the dry loading of bamboo was not significant and was a minor factor. The impregnation pressure was the major factor influencing wet loading and dry loading, which may relate to the fact that the main solvent of the agent is propylene glycol, which has a relatively high concentration and slowly enters the inner space of the bamboo and evaporates out relatively slowly during the drying process.

Based on the above analysis, it was found that tea polyphenol-modified citral was the best method to impregnate bamboo with a pressure of 0.6 MPa, an impregnation time of 150 min, and a tea polyphenol-modified citral concentration of 200 mg mL^−1^.

### 2.2. UV-Vis Analysis of Tea Polyphenol-Modified Citral Anti-Mildew-Treated Bamboo

**Figure 1:**
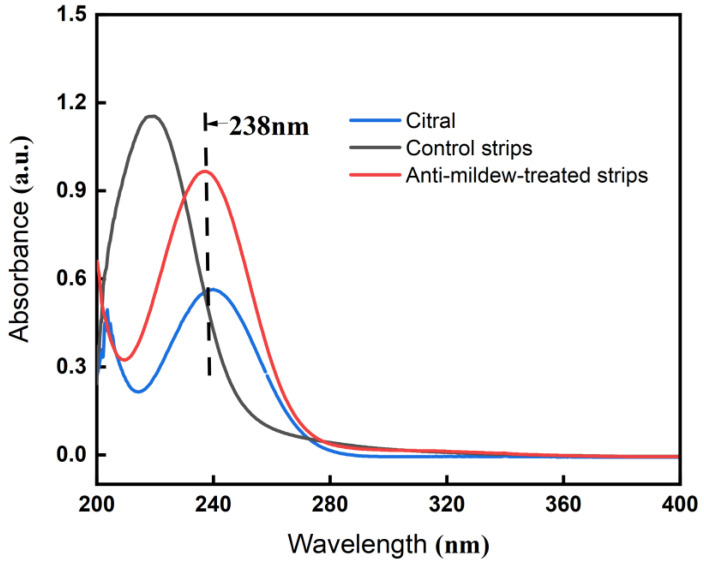
UV-vis plots of bamboo strips before and after treatment with tea polyphenols modified with citral.

Bamboo strips (referred to as anti-mildew-treated bamboo strips) treated with modified citral anti-mildew polyphenols were compared to citral-treated bamboo strips (referred to as control bamboo strips), and the UV absorbance spectra were obtained by scanning the UV band using a UV-vis spectrophotometer (UV-1800). The results are presented in Figure 1, and the changes in the UV absorbance peaks following treatment were analyzed.

Based on the spectrum of the bamboo strips with tea polyphenol-modified citral against mold in Figure 1, it can be seen that the characteristic absorption peak of citral appeared at the UV wavelength of 238 nm, which indicates that the tea polyphenol-modified citral successfully entered the bamboo material after the pressure impregnation treatment and vacuum drying, thus making the citral effective in the subsequent experiments. From the spectrum of the control-treated bamboo strips, it can be seen that the characteristic absorption peak of citral did not appear at the UV absorption wavelength of 238 nm, which indicates that the control-treated bamboo strips did not contain citral. The reason for displaying the characteristic absorption peak of citral in the UV absorption spectrum is primarily due to the good solubility of anhydrous ethanol for all substances in the tea polyphenol-modified citral, and these substances do not interfere with the UV absorbance.

### 2.3. XRD Analysis of Tea Polyphenol-Modified Citral Anti-Mildew-Treated Bamboo

**Figure 2:**
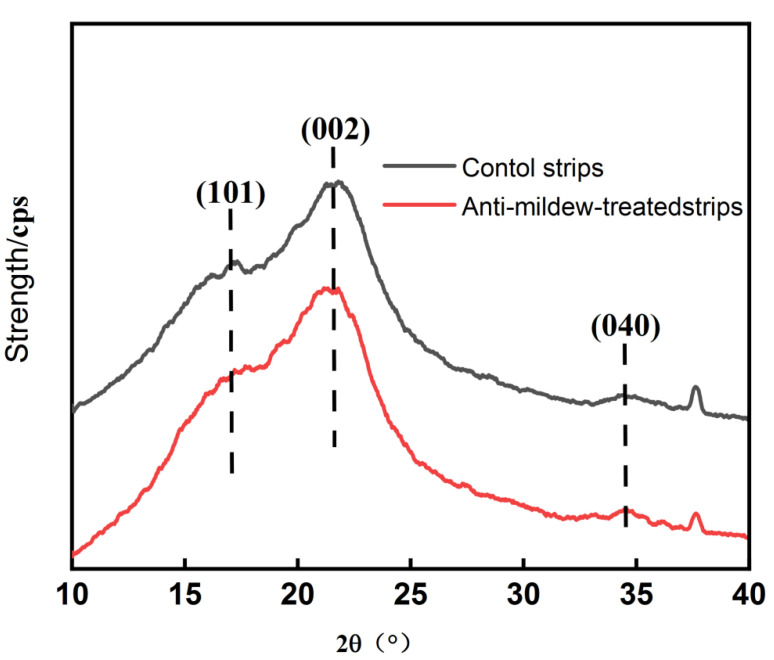
XRD plots of bamboo strips before and after impregnation treatment with tea polyphenol-modified citral.

The bamboo strips treated with the tea polyphenol-modified citral solution against mildew and the control bamboo strips were analyzed by X-ray powder diffraction, and the spectra are presented in Figure 2.

From Figure 2, we can see that the diffraction angles of peaks 101, 002, and 040 of the tea polyphenol-modified citral anti-mildew-treated bamboo strips and the control bamboo strips were around 16.54°, 21.79°, and 34.67°, which are very typical characteristic peaks of cellulose Ⅰ crystals. Of these, the (002) peaks of the control bamboo strips and the anti-mildew-treated bamboo strips were similar, that is, the cell parameters and crystalline plane spacing in the crystalline region were essentially unchanged. These results indicate that the original crystalline structure of the bamboo was not destroyed after the impregnation treatment with tea polyphenol-modified citral.

### 2.4. FT-IR Analysis of Tea Polyphenol-Modified Citral Anti-Mildew-Treated Bamboo

**Figure 3:**
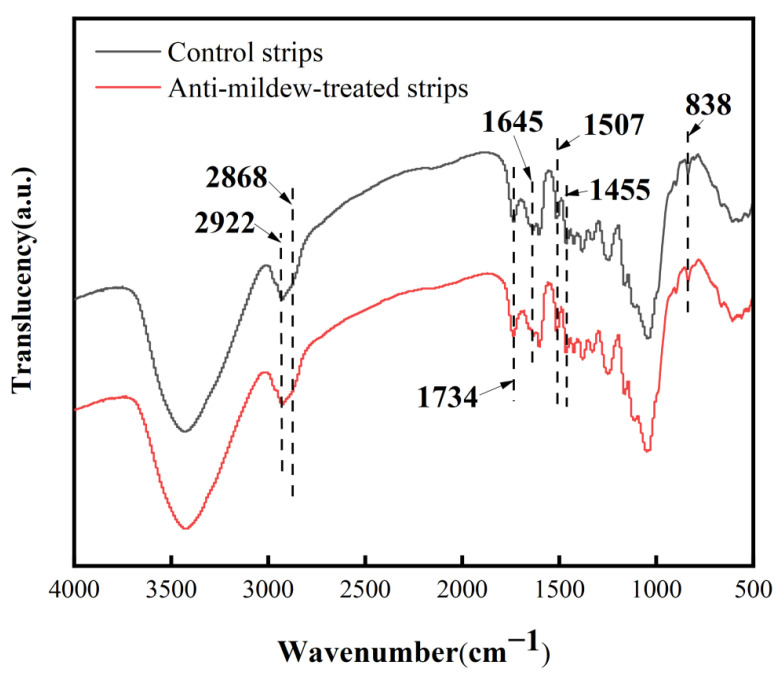
FT-IR plots of bamboo strips before and after treatment with tea polyphenol-modified citral.

The bamboo strips were impregnated with the improved anti-mildew treatment of tea polyphenol-modified citral obtained in the previous study, then dried under a vacuum, milled into bamboo powder, and compared with the control bamboo strips milled into bamboo powder and analyzed by Fourier infrared spectroscopy (FT-IR). Their infrared spectra are shown in Figure 3.

From the infrared spectra in Figure 3, it can be seen that 2922 cm^−1^ is predominantly the absorption peak of saturated -CH_3_, producing an antisymmetric stretching vibration, with 2868 cm^−1^ being the absorption peak of saturated -CH_2_, producing a symmetric stretching vibration. Here, the absorption peak of the bamboo strips after the anti-mildew treatment with tea polyphenol-modified citral is enhanced, which could be caused by the absorption of these two molecular groups in the tea polyphenol-modified citral. The absorption peak at 1734 cm^−1^ is primarily the absorption of the C=O bond, and the intensity of this peak in the anti-mildew-treated bamboo strips is enhanced significantly, which is due to the absorption of C=O in the tea polyphenol-modified citral into the bamboo material, which subsequently increases the absorption peak. The absorption peaks at 1643 cm^−1^, 1507 cm^−1^, and 1455 cm^−1^ are generated by the skeleton stretching vibration of the benzene ring. The absorption peaks here are related to the oxidative degradation of lignin and some of the citral, and the absorption peaks here are increased after the anti-mildew treatment. The 838 cm^−1^ peak is the characteristic peak of cellulose in bamboo, indicating that the chemical composition of the bamboo is not altered by the entry of tea polyphenol-modified citral into the bamboo. In summary, from the infrared spectroscopy analysis, it can be concluded that the tea polyphenol-modified citral anti-mildew treatment of bamboo strips does not alter the compositional structure of the bamboo material, and citral and some of the oxidative degradation groups can be found within the bamboo strips.

### 2.5. Anti-Mildew Properties of Tea Polyphenol-Modified Citral-Treated Bamboo

**Figure 4:**
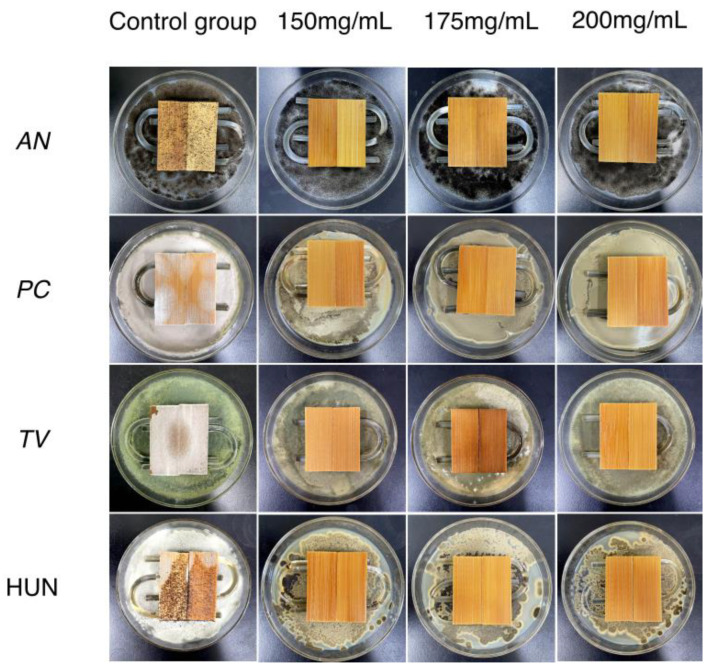
Anti-mold effects of different concentrations of the tea polyphenol-modified citral anti-mildew treatment of bamboo strips on the 28th day. *AN* (*Aspergillus niger*), *PC* (*Penicillium citrinum*), *TV* (*Trichoderma viride*), HUN (mixed molds).

**Figure 5:**
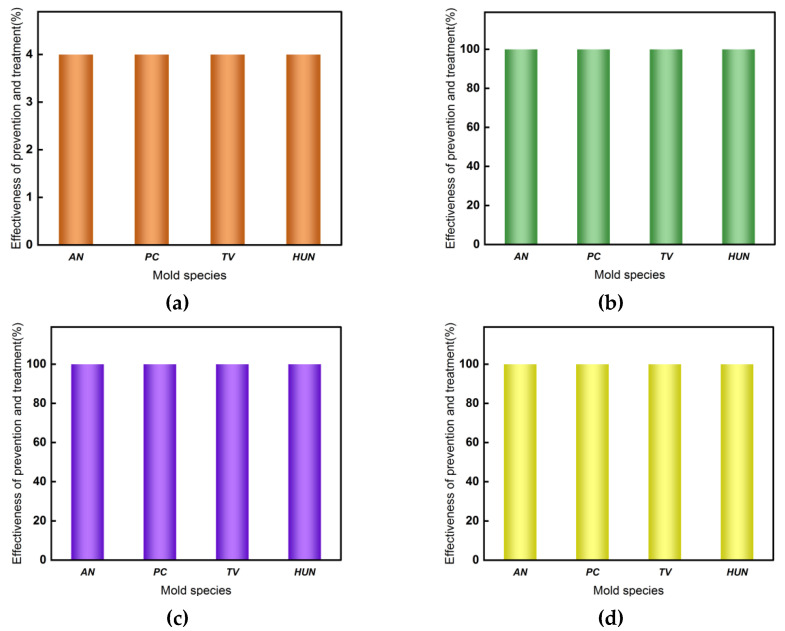
Efficacy of different concentrations of tea polyphenol-modified citral in the control of mildew on bamboo strips on day 28. (**a**) Control; (**b**) tea polyphenol-modified lemon concentration 150 mg mL^−1^; (**c**) tea polyphenol-modified lemon concentration 175 mg mL^−1^; (**d**) tea polyphenol-modified lemon concentration 200 mg mL^−1^.

The previous, better treatment has shown that the optimal concentration of tea polyphenol-modified citral is 200 mg mL^−1^, and an earlier study by the authors’ team [13] demonstrated that at a concentration of 200 mg mL^−1^, the efficacy of citral against the common molds of the bamboo material reached 100% in the anti-mildew treatment of bamboo strips. On the other hand, can the efficacy of the control of the tea polyphenol-modified citral anti-mildew treatment of bamboo at a concentration of 200 mg mL^−1^ reach 100%? This remains to be investigated. However, the 200 mg mL^−1^ concentration of tea polyphenol-modified citral for the bamboo mildew control treatment is still too high, thus increasing the cost of the mildew control treatment accordingly. Thus, without reducing the effectiveness of the control, minimizing the concentration of the tea polyphenol-modified citral for the mildew treatment of bamboo is an effective way to reduce the cost of mildew treatment. To this end, the effects of different concentrations (150 mg mL^−1^, 175 mg mL^−1^, and 200 mg mL^−1^) of the tea polyphenol-modified citral impregnation treatment of bamboo for mildew control was investigated, and the results are shown in Figure 4 and Figure 5, respectively.

From Figure 4 and Figure 5a, it can be seen that the surface of the bamboo strips in the control group had been covered with *AN*, *PC*, *TV,* and Hun, all of which had been covered in the same material. Of these, after 28 days of incubation, their infection values all reached 4.0 such that the bamboo strips in the control group had no anti-mildew effect, and it is therefore necessary to treat the bamboo against mold in order to increase its own value.

Figure 4 shows that the bamboo strips treated with different concentrations of tea polyphenol-modified citral did not get infected with *AN*, *PC*, *TV,* and Hun on their surface. When the concentration of tea polyphenol-modified citral was 150 mg mL^−1^, that is, the dry loading of bamboo strips was 22.46 g/m^2^, the lower surface of the treated bamboo strips appeared to have a very small amount of mycelium in contact with the U-shaped glass rod after 28 days of incubation; when the concentration of tea polyphenol modified-citral was 200 mg mL^−1^, that is, the dry load of the bamboo strips was 24.96 g/m^2^, the treated bamboo strips had no mycelium or mycorrhizal plates on all surfaces. From Figure 5b, it can be seen that when the concentration of tea polyphenol-modified citral was 150 mg mL^−1^, the efficacy of the treated bamboo strips against all four species reached 100% prior to day 21; however, by day 28, the effectiveness of the control against *TV* and Hun was at 100%, whereas the efficacy against *AN* and *PC* was only 90% and 92.5%, respectively. When the concentration of tea polyphenol-modified citral was 175 mg mL^−1^ and 200 mg mL^−1^, the surfaces of the treated bamboo strips were not infected by *AN*, *PC*, *TV,* and Hun. That is, the effectiveness of bamboo strips treated with a dry load of 23.77 g/m^2^ or greater against various molds reached 100% after day 28. Meanwhile, the bamboo strips had an aroma of fresh lemon. This indicates that the bamboo strips obtained by pressure impregnation with tea polyphenol-modified citral have a better control effect on common bamboo molds (*AN*, *PC*, *TV,* and Hun), so the bamboo strips treated with tea polyphenol-modified citral pressure impregnation can significantly improve the anti-mildew properties of bamboo.

## 3. Materials and Methods

### 3.1. Materials

#### 3.1.1. Bamboo

Bamboo strips made from Moso bamboo were processed into 50 mm × 20 mm × 5 mm (length × width × thickness) specimens with no bamboo knots and with a moisture content of about 10%. The bamboo strips were purchased from Zhejiang Yongyu Bamboo Industry Co. (Huzhou, China).

#### 3.1.2. Main Reagents

Citral (cis-trans isomer mixture), 97% propylene glycol, anhydrous ethanol, agar powder, and glucose, all of which were analytically pure, were purchased from the Sinopharm Chemical Reagent Co., Ltd. (Shanghai, China).

#### 3.1.3. Test Strains

*Penicillium citrinum* (*PC*), *Trichoderma viride* (*TV*), *Aspergillus niger* (*AN*), and mixed molds (HUN, mixed by *PC*, *TV,* and *AN* in equal proportions).

### 3.2. Methods

#### 3.2.1. Preparation of Tea Polyphenol-Modified Citral

A suitable amount of citral was added to propylene glycol at room temperature, and then the mixture was placed in a magnetic collecting stirrer and stirred until the citric acid dissolved. Anhydrous ethanol and deionized water were then added and brewed, and then polyphenols and tea citral were added in turn and brewed for some time to make the citral-modified tea polyphenols. Tea polyphenol was added at a concentration of 1% of the citral mass.

#### 3.2.2. Tea polyphenol-Modified Citral Impregnation Treatment of Bamboo

A certain number of bamboo strips were weighed in groups (recorded as m_1_), and then one group was soaked in a beaker containing a tea polyphenol-modified citral solution, and the other group was soaked in a beaker containing a citral solution as a control group. Next, all the beakers were placed in a pressurized tank and vacuumed to 0.085 MPa. The vacuum was maintained for 30 min. At the end, the outlet valve was opened, and the valve was closed when the beaker returned to a normal pressure. The bamboo was pressurized and impregnated under the conditions set by the test. Next, the treated bamboo flakes (recorded as m_2_) were removed and weighed, then placed in a vacuum dryer, and dried at 30℃ until about 10% water content remained. Again, they were removed and weighed (recorded as m_3_). The bamboo chips were then placed in a dry dish in a sealed bag for subsequent anti-mold treatment.

#### 3.2.3. Experimental Design of the Tea Polyphenol-Modified Citral Anti-Mildew Treatment of Bamboo

Pressure, impregnation time, and concentration of tea polyphenol-modified citral were used as the influencing factors, and three orthogonal test levels of L_9_ (3^4^) were chosen to perform the test of the tea polyphenol-modified citral impregnation treatment of bamboo strips. The table of test factor levels is given in Table 4. The wet load (R_1_) and dry load (R_2_) of the bamboo material were used as the judgment indexes to determine the better impregnation treatment process and the results of R_1_ and R_2._ For orthogonal experiments, ANOVA was performed. In each case, six bamboo strips were treated with the same trial number, each trial number was repeated two times, and the results were averaged. 

The wet loading (R_1_) and dry loading (R_2_) of the bamboo were calculated using Equation (1) and Equation (2), respectively:

(1) $$R_{1}=\frac{\left(m_{2}-m_{1}\right)\times c\times 10^{6}}{s}$$

(2) $$R_{2}=\frac{\left(m_{3}-m_{1}\right)\times c\times 10^{6}}{s}$$

where R_1_ and R_2_ are the wet load and dry load, respectively, in g/m^2^; m_1_ is the mass of the bamboo strip before treatment in g; m_2_ is the wet mass of the bamboo strip after treatment in g; m_3_ is the mass of the bamboo strip after treatment and drying in g; c is the mass fraction of tea polyphenol-modified citral in %; and s is the sum of the 6 bamboo strip surfaces in m^2^.

#### 3.2.4. Ultraviolet-Visible Spectrophotometer (UV-Vis) Analysis of Tea Polyphenol-Modified Citral Anti-Mildew-Treated Bamboo

The bamboo strips impregnated with tea polyphenol-modified citral were taken out in the appropriate amount, powdered and dissolved in anhydrous ethanol, sonicated for 15 min, placed in a centrifuge, and centrifuged at 9000 r/min for 10 min; the supernatant was then placed in a quartz cuvette and scanned using a UV-visible spectrophotometer (UV-1800) in the wavelength range of 200 to 300 nm in order to analyze changes in the UV absorption spectra of the bamboo strips with different treatments.

#### 3.2.5. X-Ray Diffraction (XRD) Analysis of Tea Polyphenol-Modified Citral Anti-Mildew-Treated Bamboo

The X-ray tube was a copper target, the tube voltage was 40 kV, the tube current was 30 mA, and the scan range of the time was 10°~40° (2θ) with a speed of 2°/min.

#### 3.2.6. Fourier Transform Infrared Spectroscopy (FT-IR) Analysis of Tea Polyphenol-Modified Citral Anti-Mildew-Treated Bamboo

The impregnated treated bamboo strips were dried under a vacuum, ground into bamboo powder using a micro plant grinder, screened using a 300-mesh sieve, mixed well with the powder mass of potassium bromide according to 1:100, squeezed into thin slices, and ultimately the molecular structure of the bamboo strips was analyzed by the Fourier transform infrared (FT-IR) IR Prestige-21 spectrometer. The FT-IR analysis had a resolution of 4 cm^−1^ and a wavelength range of 4000~500 cm^−2^. All samples were scanned 3 times, and spectra were required to appear at least 2 overlapping times.

#### 3.2.7. Preparation of Potato Glucose Agar Medium

First, 400 g of peeled potato was weighed, boiled with an appropriate quantity of water for 30 min, and filtered through two layers of gauze to yield the filtrate; the filtrate was then supplemented with 40 g of glucose and 48 g of agar, and water was added to fix the volume to 2000 mL, and the mixture was agitated until melting of the agar. The potatoes were finally divided into three wide-mouth conical vials, sealed with a high temperature sealing film, and placed in an autoclave at 121 °C and 0.1 MPa for sterilization for 1 h. The potato glucose agar media plate was generated for the subsequent cultivation of the strain.

#### 3.2.8. Preparation of Bacterial Suspension

A wide-mouth bottle was placed with an appropriate amount of deionized water and small glass beads in the autoclave and sterilized at 121 °C and 0.1 MPa for 1 h. Then, it was inoculated on the biological clean table (sterile environment). The mycelium and spores of the test strain were picked with the inoculation needle, then placed in the sterilized wide-mouth flask, and finally shaken for 10 min~15 min to produce the mycobacterial suspension for inoculation.

#### 3.2.9. Experiment on the Tea Polyphenol-Modified Citral Anti-Mildew Treatment of Bamboo

In accordance with the Chinese national standard test method “experimental method for the effectiveness of anti-mildew agents against wood mold and its discoloration bacteria” (GB/T 18261-2013) [27], four strain suspensions of *Trichoderma viride* (*TV*), *Penicillium citrinum* (*PC*), *Aspergillus niger* (*AN*), and mixed molds (the strain suspension ratio of *TV*, *PC,* and *AN* is 1:1:1, denoted as HUN) were evenly coated onto the plate medium in the Petri dishes, and sterilized U-shaped glass rods were placed on top. The bamboo strips to be experimented on and the control group blank bamboo strips were placed on the glass rods and were finally sealed with a piece of sterile sealing film from the edge of the Petri dishes and were placed in the artificial climate chamber to maintain the temperature at 28 ± 2 ℃ with 85 ± 5% relative humidity. The degree of mold-infected bamboo strips in the climate chamber was observed and recorded on a daily basis during the experimental period (judged by Table 5), and the effectiveness of the bamboo strips against mold was calculated based on Equation (3). Each group was replicated three times to determine the degree of infection in the bamboo strips for each mold, and the results of the mold prevention experiment were analyzed.

The equation for the prevention and treatment effectiveness is as follows:

(3) $$E=(1-\frac{D_{1}}{D_{0}})\times 100\%$$

In the formula, E: control efficacy, %; D_1_: average infection value of bamboo strips treated with antioxidant citral, and D_0_: average infection value of untreated bamboo strips in the control group.

## 4. Conclusions

1. The better treatment parameters of the tea polyphenol-modified citral anti-mildew treatment of bamboo are as follows: impregnation pressure 0.6 MPa, impregnation time 150 min, and tea polyphenol-modified citral concentration 200 mg mL^−1^, where the impregnation pressure has a significant effect on the dry and wet loading of anti-mildew-treated bamboo.
2. Tea polyphenol-modified citral does not react with the chemical composition of bamboo after anti-mildew treatment and does not destroy the original microstructure and chemical form of the bamboo.
3. When the concentration of tea polyphenol-modified citral reached 175 mg mL^−1^ and above, the efficacy of its anti-mildew treatment on common bamboo materials such as *PC*, *TV*, *AN,* and Hun was found to be 100% effective; the tea polyphenol-modified citral anti-mildew treatment on bamboo materials was found to have better anti-mildew properties.
4. Following treatment with tea polyphenol-modified citral, bamboo wood had significantly improved its anti-mold performance and added a refreshing lemon fragrance. Thus, tea polyphenol modified-citral has a good application prospect in the anti-mildew treatment of bamboo.

## Figures and Tables

**Table 1:** Table of orthogonal experiments and results.

Experimental Number	Factors			R_1_ (g/m^2^ )	R_2_ (g/m^2^ )
	A (Pressure/MPa)	B (Impregnation Time/min)	C (Concentration mg mL^−1^)		
1	1	1	1	26.80	11.92
2	1	2	2	28.89	14.46
3	1	3	3	30.42	16.27
4	2	1	2	33.69	19.86
5	2	2	3	37. 19	23.42
6	2	3	1	35.58	22.46
7	3	1	3	34.25	20.05
8	3	2	1	31.30	18.04
9	3	3	2	36.82	20.85
X1 (R1)	28.703	31.580	31.227		
X2 (R1)	35.487	32.460	33. 133		
X3 (R1)	34.123	34.273	33.953		
R (R1)	6.784	2.693	2.726		
X1 (R2)	14.217	17.277	17.473		
X2 (R2)	21.853	18.580	18.390		
X3 (R2)	19.647	19.860	19.853		
R (R2)	7.636	2.583	2.380		

R = X_max_ − X_min_.

**Table 2:** Analysis of variance for the wet drug load R_1_.

Sources of Variance	Sum of Squares of Deviations	Degrees of Freedom	F-Value	F_0.1_ (2, 2)	Significance
A	77.249	2	15.628	9	*
B	11.317	2	2.290	9	
C	11.742	2	2.375	9	
Error	4.94	2			
Sum	105.248	8			

“*” Indicates significant difference.

**Table 3:** Analysis of variance (ANOVA) for R_2_ of dry drug loading.

Sources of Variance	Sum of Squares of Deviations	Degrees of Freedom	F-Value	F_0.1_ (2, 2)	Significance
A	92.673	2	68.647	9	*
B	10.011	2	7.416	9	
C	8.646	2	6.404	9	
Error	1.35	2			
Sum	112.68	8			

“*” Indicates significant difference.

**Table 4:** Table of experimental factor levels.

Level		Factors	
	A (Pressure/MPa)	B (Impregnation Time/min)	C (Concentration mg mL^−1^)
1	0.5	90	150
2	0.6	120	175
3	0.7	150	200

**Table 5:** Grading of the surface infection values of the bamboo strips subjected to mold.

Infection Value	Area Infected with Bamboo Strips
0	No mycelium or mold on the surface of the bamboo strips
1	Infected area of bamboo strips surface < 1/4
2	Infected area of bamboo strips surface 1/4–1/2
3	Infected area of bamboo strips surface
4	Infected area of bamboo strips surface > 3/4

## Data Availability

Not applicable.
